# Diagnostic and Prognostic Value Analysis of miR-206 in Asymptomatic Carotid Artery Stenosis

**DOI:** 10.3389/bjbs.2022.10592

**Published:** 2022-07-06

**Authors:** Dancen Li, Jingjun Pan

**Affiliations:** ^1^ Department of Neurosurgery, Changzhou No. 2 People’s Hospital, Changzhou, China; ^2^ Intensive Care Unit, Changzhou No. 2 People’s Hospital, Changzhou, China

**Keywords:** prognosis, diagnosis, carotid artery stenosis, miR-206, miRNA

## Abstract

**Introduction:** To investigate the expression level of miR-206 in serum of patients with asymptomatic carotid artery stenosis (CAS) and estimate the value of miR-206 in the diagnosis and prognosis of asymptomatic CAS.

**Methods:** A total of 206 individuals enrolled in this study, including 105 CAS patients and 101 controls. RT-qPCR technology was applied to measure the relative level of miR-206, and Pearson’s correlation coefficient was performed to analyze the relationship between carotid artery stenosis degree and miR-206 level. An ROC curve was drawn to assess the diagnostic value of miR-206 in asymptomatic CAS. The 5-year prognosis of asymptomatic CAS patients was tested using multivariate Cox regression analysis and Kaplan-Meier survival curve.

**Results:** MiR-206 expression was reduced in asymptomatic CAS patients. The AUC of the ROC curve of miR-206 was 0.939, with a sensitivity of 86.70% and a specificity of 86.14%. The amount of CAS gradually increased with the decrease of miR-206 level. Seven-teen patients in the low miR-206 expression group developed CIEs, and 3 patients in the high miR-206 expression group developed CIEs during the 5-year follow-up. miR-206 and the amount of CAS were independent factors for the occurrence of CIEs within 5 years in asymptomatic CAS patients.

**Conclusion:** Serum miR-206 has high diagnostic accuracy for asymptomatic CAS and has predictive value for the incidence of CIEs in patients within 5 years.

## Introduction

Cardiovascular and cerebrovascular diseases have become the third leading cause of death in China, and they bring a heavy economic burden to families and society [1]. The clinical symptoms of carotid artery stenosis [2] may manifest as symptoms related to ischemic stroke, or may not produce any clinical symptoms, and these mainly depend on whether the collateral circulation of stenotic vessels can be compensated in time and the stability of plaques in stenotic areas [3]. When CAS is not compensated in time due to collateral circulation or plaque unstablity, it will lead to memory and orientation loss, disturbance of consciousness, aphasia, blindness, numbness of limbs and dizziness. Patients with severe CAS may have dementia, transient ischemic attack, and even ischemic cerebral infarction. Conversely, when the collateral circulation of stenotic vessels is well compensated or the plaque is stable enough, it may not cause any obvious clinical symptoms, that is, asymptomatic CAS [4]. In recent years, with the aging population growing, asymptomatic CAS has become of increasing importance.

At present, the clinical methods used for CAS diagnosis are mainly imaging examinations, including carotid artery ultrasound and CT angiography [5, 6]. However, due to the lack of typical clinical manifestations in asymptomatic CAS patients at the early stage and the high cost of imaging diagnosis, it would be useful to find other technical and biological markers to help in early clinical detection of CAS. MicroRNA (miRNA) plays an important role in gene epigenetic regulation [7]. miRNAs are abundant in eukaryotes, and they can tolerate unfavorable conditions such as strong acids and alkalis to exist stably in the blood [8, 9]. Studies have shown that miRNAs can reduce the formation of foam cells by reducing lipid formation in macrophages, and can also regulate atherosclerosis by affecting the production of inflammatory factors [10]. Loyer et al. found that miR-92a is highly expressed in vascular endothelial cells in the presence of oxidized low-density lipoprotein, which in turn increases the damage of ox-LDL to endothelial cells [11]. Harris et al. found that miR-126a is related to vascular inflammation, and it can promote the aggregation of white blood cells under endothelial cells [12]. Human miR-206 is located on chromosome 6 and is a member of miR-1 family with specific muscle expression [13]. One study reported that the expression of miR-206 in atherosclerotic tissue samples decreased [14], and another reported that miR-206 level in the renal artery of hyperlipidemia rats decreased, which was closely related to the increase of renal artery hyperreactivity [15]. Atherosclerosis, hyperlipidemia, and other factors are the inducement and risk factors of CAS, and we hypothesised that miR-206 may be abnormally expressed in asymptomatic CAS.

In the present study, relevant data of miR-206 in the serum of the target subjects were tested through a single-center clinical experiment and data analysis technique, and the diagnostic and prognostic value of miR-206 in asymptomatic CAS was appraised.

## Materials and Methods

### Objects of Study

A total of 105 asymptomatic CAS admitted to this hospital were identified as the case group. Patients with previous history of stroke and transient ischemic attack, and patients with heart failure and congestive heart failure were excluded. Another 101 outpatients were chosen as the control group, and CAS was excluded, including 46 males and 55 females, with an average age of (62.67 ± 10.26) years. This project has passed the examination and approval of Changzhou No. 2 People’s Hospital Ethics Committee and informed consent was obtained from all subjects. Demographic characteristics of all subjects were recorded and analyzed, including blood pressure, blood sugar, blood lipid and other clinical indicators, as well as the number of patients with diabetes, hypertension, and hyperlipidemia. Hypertension was defined as systolic blood pressure (SBP) ≥140 mmHg and/or diastolic blood pressure (DBP) ≥ 90 mmHg. Hyperlipidemia was defined as serum total cholesterol ≥5.17 mmol/L (200 mg/dl), and/or serum triglycerides ≥1.69 mmol/L (150 mg/dl). Additionally, for this study, smoking history was defined as cumulative smoking of more than 100 cigarettes to date. Drinking history was defined as drinking more than once a week, and the amount of drinking was more than 2 standard amounts for men and 1 standard amount for women. A standard amount referred to alcoholic beverages containing 12 g of ethanol.

### Determination of CAS

The left and right carotid arteries of the subjects were examined by vivid 7 color Doppler ultrasound diagnostic instrument (GE Company, USA), and the stenosis degree of carotid artery was determined according to the carotid ultrasound area method and the standard of the North American Symptomatic Carotid Endarterectomy Trial (NASCET) [16].

### Sample Collection

In this study, blood samples were collected from the control group and carotid artery stenosis group. Collect 5 m L venous blood in the morning after admission. After centrifugation, the upper serum was taken and placed in EP tube and immediately stored in -80 cryogenic refrigerator for inspection.

### Determination of Serum miR-206 Expression Levels

The serum miR-206 level was determined by RT-qPCR analysis. Total RNA in serum was separated by Trizol reagent, and the concentration and purity of RNA were detected by UV spectrometer. When the ratio of OD260/OD280 is in the range of 1.8–2.0, it means that the RNA purity is qualified. cDNA was synthesized using a reverse transcription kit and then amplified according to the instructions on the PCR kit. PCR results were obtained by 2^−ΔΔCT^ method.

### Follow up Analysis

Asymptomatic CAS patients were grouped into miR-206 high level group and miR-206 low level group according to the median level of miR-206. A 5-year telephone follow-up study was performed on all asymptomatic CAS patients, and the number of cerebral ischemic events (CIEs) such as transient ischemic attack (TIA), stroke, and sudden death occurred in all patients during the 5-year period were recorded.

### Statistical Analysis

Graphpad Prism 6.0 and SPSS 17.0 software are used for data processing and analysis. The quantitative data were manifested as mean ± SD, and the comparison between groups was made by one-way ANOVA. The qualitative data were represented by n, and the chi-square test was used for comparison between two groups. The diagnostic value of miR-206 was estimated by an ROC curve. Correlation analysis was conducted by Pearson’s test. Cox regression and Kaplan-Meier survival curve were applied to assess the prognosis of CAS patients.

## Results

### Comparisons of Baseline Data

Table 1 shows the clinical information and medical history of asymptomatic CAS patients and non-CAS patients recruited for this study. There were no significant differences in age, gender, BMI, smoking and drinking history, diabetes, hyperlipidemia, hypertension, and other pathological indicators between the two groups (*p* > 0.05), while DBP and TG values in CAS group were higher than those in CAS group (*p* < 0.05). Besides, imaging results showed that the carotid artery diameter of asymptomatic CAS patients was reduced by about (67.17 ± 9.38)%.

**TABLE 1:** Comparison of clinical data of patients in the study.

Items	Non-CAS group (*n* = 101)	CAS group (*n* = 105)	Significant (p)
Age (years)	62.7 ± 10.3	61.7 ± 9.7	0.470
Male (n, %)	46 (45.6)	55 (52.4)	0.326
BMI (kg/m^2^)	20.4 ± 3.0	21.1 ± 2.9	0.078
Diabetes (n, %)	45 (44.6)	57 (54.3)	0.163
Hyperlipidemia (n, %)	63 (62.4)	73 (69.5)	0.279
Hypertension (n, %)	45 (44.6)	58 (55.2)	0.125
Medication			0.692
Statins	32 (31.7)	50 (47.6)	
ACEIs	23 (22.8)	42 (40.0)	
Antidiabetic drugs	29 (28.7)	39 (37.1)	
SBP (mmHg)	129.57 ± 14.15	135.88 ± 17.07	0.276
DBP (mmHg)	76.13 ± 10.54	85.64 ± 13.73	0.000
TC (mmol/L)	4.95 ± 0.89	5.34 ± 1.16	0.116
TG (mmol/L)	1.36 ± 0.41	1.41 ± 0.21	0.034
HDL-C (mmol/L)	1.21 ± 0.29	1.29 ± 0.34	0.117
LDL-C (mmol/L)	2.58 ± 0.33	2.62 ± 0.28	0.202
FBG (mmol/L)	6.47 ± 1.68	6.61 ± 1.82	0.653
HbA1c (%)	6.35 ± 1.13	6.55 ± 1.20	0.251
Smoking (n, %)	47 (46.5)	51 (48.6)	0.578
Drinking (n, %)	45 (44.6)	53 (50.5)	0.395
Degree of carotid artery stenosis	—	67.17 ± 9.38	—

Abbreviations: CAS, carotid artery stenosis; BMI, body mass index; SBP, systolic blood pressure; DBP, diastolic blood pressure; TC, total cholesterol; TG, triglyceride; HDL-C, high-density lipoprotein cholesterol; LDL-C, low-density lipoprotein cholesterol; FBG, fasting blood glucose. HbA1c, Glycosylated Hemoglobin, Type A1C. Data are expressed as n or mean ± standard deviation.

### MiR-206 Was Declined in Asymptomatic CAS Patients and Showed High Diagnostic Accuracy for CAS

**FIGURE 1:**
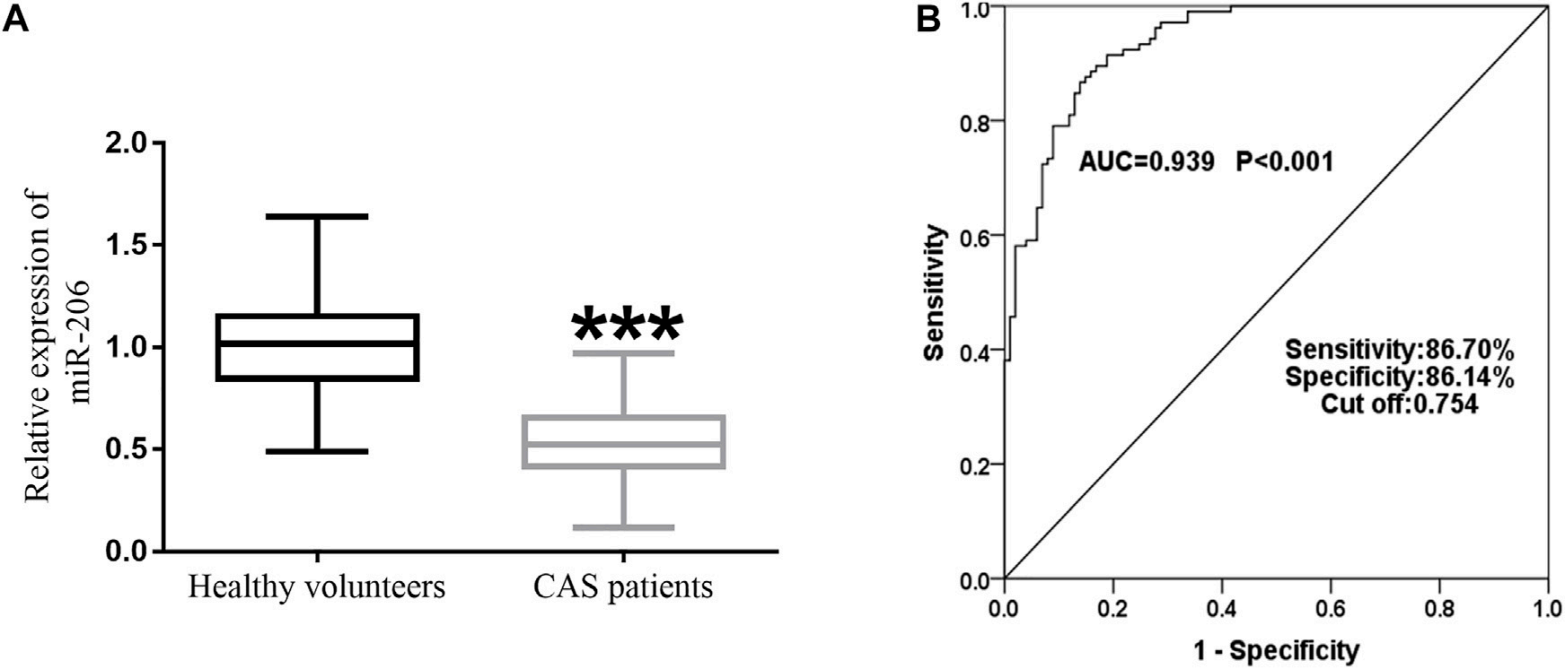
Analysis of miR-206 expression level and diagnostic value for asymptomatic CAS. **(A)** RT-qPCR showed that serum miR-206 expression was reduced in asymptomatic CAS patients (*p* < 0.001). **(B)** The AUC of miR-206 was 0.939, and the sensitivity and specificity were 86.70% and 86.14%, respectively.

Decreased expression of miR-206 was detected in asymptomatic CAS patients compared to miR-206 levels in non-CAS patients (Figure 1A, *p* < 0.001). Figure 1B shows the ROC curve of miR-206. It was found that when the cutoff value was 0.754, the AUC of miR-206 was 0.939, and the sensitivity and specificity were 86.70% and 86.14%, respectively, which indicated that miR-206 had good diagnostic accuracy for asymptomatic CAS.

### The Degree of Carotid Stenosis Increased With the Decrease of miR-206

**FIGURE 2:**
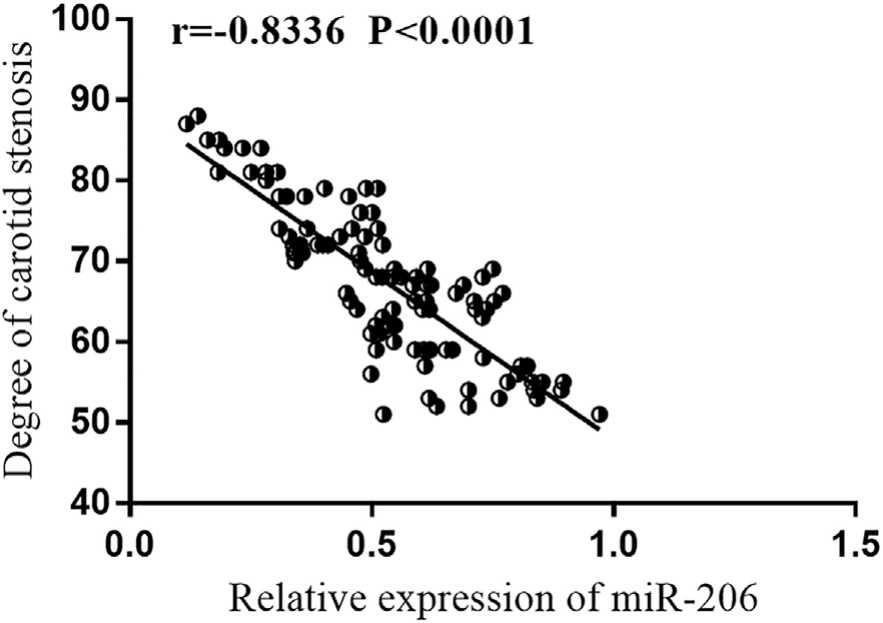
Pearson correlation coefficient analysis. Pearson’s correlation analysis showed that the degree of carotid artery stenosis was negatively correlated with the expression level of miR-206.

AS shown in Figure 2, Pearson’s correlation coefficient analysis showed that the degree of carotid stenosis was negatively correlated with the level of miR-206. That is to say, the lower the level of miR-206, the higher the degree of carotid stenosis (r = −0.8336, *p* < 0.0001). Subsequently, multivariate logistic regression analysis indicated that miR-206 was closely related with the degree of carotid stenosis (Table 2, OR = 2.977, 95% CI = 1.167–7.595, *p* = 0.022).

**TABLE 2:** Association of different variables with the occurrence of carotid artery stenosis.

Variables	OR	95% CI	*p* value
MiR-206	0.336	0.132–0.857	0.022
Age	0.450	0.167–1.211	0.114
Gender	0.695	0.278–1.737	0.437
BMI	1.175	0.467–2.956	0.732
Diabetes	1.408	0.508–3.909	0.511
Hyperlipidemia	1.127	0.409–3.107	0.817
Hypertension	0.804	0.303–2.133	0.661
Smoking	0.548	0.212–1.417	0.214
Drinking	1.504	0.585–3.870	0.397
SBP	0.673	0.164–2.756	0.582
DBP	0.261	0.161–1.112	0.069
TC	1.158	0.455–2.944	0.758
TG	0.445	0.164–1.208	0.112
HDL	0.868	0.333–2.263	0.772
LDL	0.653	0.254–1.680	0.376
HbA1c	1.371	0.516–3.644	0.526
FBG	0.495	0.152–1.619	0.254
Medication	2.169	0.666–7.706	0.199

Abbreviations: BMI, body mass index; SBP, systolic blood pressure; DBP, diastolic blood pressure; TC, total cholesterol; TG, triglyceride; HDL-C, high-density lipoprotein cholesterol; LDL-C, low-density lipoprotein cholesterol; FBG, fasting blood glucose. HbA1c, Glycosylated Hemoglobin, Type A1C.

### Patients With low Expression of miR-206 Were More Likely to Have CIEs Within 5 Years

**FIGURE 3:**
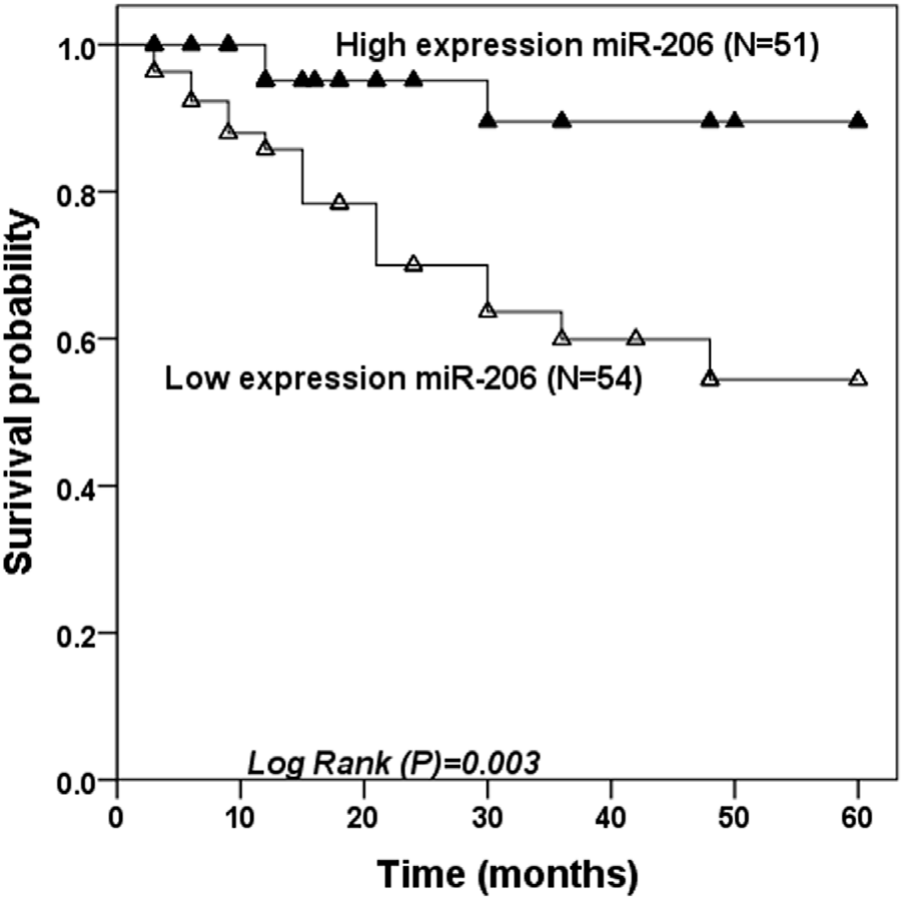
Kaplan Meier survival analysis. A 5-year follow-up analysis was performed after patients in the asymptomatic CAS group were grouped based on median miR-206 values.

The K-M curve manifested that 3 patients in the miR-206 high level group developed CIEs within 5 years, including 1 TIA and 2 strokes, while 17 patients in the miR-206 low expression group developed CIEs (1 sudden death, 10 TIAs, and 6 strokes), indicating that the low miR-206 expression group had a greater chance of developing CIEs during the 5-year follow-up (Figure 3, log rank *p* = 0.003). In Table 3, multivariate Cox regression analysis investigated that down-regulated serum miR-206 (HR = 0.046, 95% CI = 0.005–0.431, *p* = 0.007) and degree of CAS (HR = 0.236, 95% CI = 0.062–0.889, *p* = 0.034) were the independent risk factors for the development of CIEs in asymptomatic CAS patients during 5-year follow-up.

**TABLE 3:** Multivariate COX analysis for MiR-206 and established risk factors.

Items	Multivariate analysis		
	HR	95% CI	*p*
MiR-206	0.046	0.005–0.431	0.007
Age	0.454	0.280–1.533	0.161
Gender	0.546	0.132–1.906	0.241
BMI	0.621	0.186–2.069	0.438
Diabetes	0.724	0.511–2.287	0.380
Hypercholesterolemia	0.573	0.391–1.678	0.429
Hypertension	0.339	0.116–1.452	0.173
Smoking	0.539	0.276–1.808	0.452
Drinking	0.716	0.245–2.289	0.578
Degree of CAS	0.236	0.062–0.899	0.034
SBP	0.422	0.215–1.334	0.188
DBP	0.647	0.297–2.142	0.385
TC	0.372	0.087–1.598	0.184
TG	0.556	0.447–1.317	0.487
HDL-C	0.734	0.204–2.646	0.637
LDL-C	0.761	0.231–2.507	0.653
FBG	0.708	0.111–2.054	0.515
HbA1c	0.512	0.353–1.738	0.109
Medication	0.324	0.073–1.435	0.138

Abbreviations: BMI, body mass index; CAS, carotid artery stenosis; SBP, systolic blood pressure; DBP, diastolic blood pressure; TC, total cholesterol; TG, triglyceride; HDL-C, high-density lipoprotein cholesterol; LDL-C, low-density lipoprotein cholesterol; FBG, fasting blood glucose. HbA1c, Glycosylated Hemoglobin, Type A1C.

## Discussion

More than two-thirds of the blood supply to the brain passes through the carotid artery [17]. In the early stages of atherosclerosis, the degree of carotid artery stenosis is mild and has little effect on blood flow through the carotid artery, so the patient will be asymptomatic (asymptomatic CAS) [18, 19]. With the severity of carotid artery stenosis increasing, carotid blood flow is reduced, resulting in insufficient blood supply to the brain, and then a series of typical clinical manifestations appear, which is then referred to as symptomatic CAS [20]. With the aging population and changing diet in China, the prevalence of CAS among middle-aged and elderly people in is gradually increasing. Due to the high cost of imaging tests, there is a need for new biological markers to assist in the diagnosis and prediction of CAS.

MiRNAs are post-transcriptional regulators that regulate gene expression by facilitating mRNA degradation or inhibiting protein synthesis [21]. MiR-206 is located on human chromosome 6p12.2 with a highly conserved sequence [22]. MiR-206 has more than 500 target genes, which are mainly involved in biological processes such as Wnt signaling and biosynthesis regulation [2, 23]. Dysregulation of miR-206 has been observed in different types of cardiovascular disease. For instance, miR-206 is abundantly expressed in endothelial progenitor cells and the plasma of patients with coronary heart disease (CHD), where there is no significant correlation between the expression level and different clinical and pathological features [24]. A clinical study found that miR-206 expression in circulating blood was decreased in patients with left-sided heart disease, which was correlated with an increase of systolic blood pressure of pulmonary artery [25]. Vascular smooth muscle cells (VSMCs) participate in plaque stabilization and vascular remodeling and play an important role in the formation of atherosclerosis [16]. Studies have shown that miR-206 expression decreases in atherosclerotic tissue samples, and further studies have shown that miR-206 reduction reduces the relative survival rate of VSMCs [14]. Furthermore, miR-206 expression is reduced in renal artery tissue from diabetic and hyperlipidemic rats. High glucose and lipid downregulate the expression of miR-206 by inducing metabolic inflammation, and ultimately induce functional changes in VSMCs [15]. Considering the abnormal expression of miR-206 in hypertension, atherosclerosis, diabetes, and hyperlipidemia, we hypothesised that it may be abnormal in CAS. In this study, the expression of miR-206 in serum of asymptomatic CAS patients was notably decreased compared with that of the control group. The subsequent ROC method displayed that the AUC value of miR-206 was 0.939, indicating that testing for miR-206 may be sufficiently sensitive to aid in the diagnosis of asymptomatic CAS and therefore have clinical value.

CAS is one of the risk factors of CIEs, and the degree of CAS is affected by many factors [26]. In our paper, it was found that the degree of CAS gradually deepened with the decrease of miR-206 level, and among many clinical factors, the expression of miR-206 was closely related to the degree of CAS. In view of the association between miR-206 and the degree of CAS, we speculated that miR-206 may have the potential CIEs predictive ability. During the 5-year follow-up analysis, it was observed that among these asymptomatic CAS patients, the incidence of CIEs in patients with low expression of miR-206 was significantly higher than that in patients with high expression of miR-206, indicating that serum miR-206 was a strong predictor of CIEs in asymptomatic CAS patients within 5 years. Multivariate Cox regression analysis confirmed that miR-206 was a risk factor for developing CIEs in asymptomatic CAS patients within 5 years.

The limitations of this study are as follows: for follow-up studies telephone follow-up may make the statistics and records of CIEs incomplete and insufficient; secondly, the diet of patients has not been deeply explored, as daily diet and exercise are factors affecting the prognosis of patients with CAS. In addition, the control group recruited in this study is not a healthy cohort, but a non-CAS population matched with the CAS group. We do not know the actual expression of miR-206 in the healthy population at present. A comparison of the difference in mir-206 expression between healthy and CAS groups will help us to have a deeper understanding of the role and significance of miR-206 in CAS.

In summary, the decreased serum expression of miR-206 in CAS patients and miR-206 level was closely related to the degree of CAS observed. Our study preliminarily shows the clinical value of miR-206 in asymptomatic CAS, and provides an evidence base for future research on CAS.

## Summary Table

### What Is Known About This Subject

• Despite a well-established clinical treatment regime, patients with carotid artery stenosis are at high risk for adverse events.
• In most hospitals, imaging diagnosis of carotid artery stenosis is expensive.
• Imaging diagnosis requires a high level of medical expertise, which can be expensive and time-consuming.

### What This Paper Adds

• Low levels of miR-206 has high sensitivity and specificity for the diagnosis of asymptomatic carotid artery stenosis.
• The degree of carotid artery stenosis increases with the decrease of miR-206 expression levels.
• The overall survival rate of patients with low miR-206 expression was less than that of patients with high miR-206 expression.

## Summary Sentence

This work represents an advance in biomedical science because it shows that assay of miR-206 levels may be useful in the diagnosis and prognosis of patients with carotid artery stenosis.

## Data Availability

The original contributions presented in the study are included in the article/supplementary material, further inquiries can be directed to the corresponding author.
